# *In vitro* evaluation of the anti-leishmanial activity and toxicity of PK11195

**DOI:** 10.1590/0074-02760170345

**Published:** 2018-02-05

**Authors:** Carlos Eduardo Sampaio Guedes, Beatriz Rocha Simões Dias, Antonio Luis de Oliveira Almeida Petersen, Kercia Pinheiro Cruz, Niara de Jesus Almeida, Daniela Rodrigues Andrade, Juliana Perrone Bezerra de Menezes, Valéria de Matos Borges, Patricia Sampaio Tavares Veras

**Affiliations:** 1 Fundação Oswaldo Cruz-Fiocruz, Centro de Pesquisas Gonçalo Moniz, Laboratório de Patologia e Biointervenção, Salvador, BA, Brasil; 2 Fundação Oswaldo Cruz-Fiocruz, Centro de Pesquisas Gonçalo Moniz, Laboratório Integrado de Microbiologia e Imunoregulação, Salvador, BA, Brasil

**Keywords:** PK11195, Leishmania amazonensis, macrophage, chemotherapy

## Abstract

**BACKGROUND:**

Leishmaniasis, one of the most neglected diseases, is a serious public health problem in many countries, including Brazil. Currently available treatments require long-term use and have serious side effects, necessitating the development of new therapeutic interventions. Because translocator protein (TSPO) levels are reduced in *Leishmania amazonensis*-infected cells and because this protein participates in apoptosis and immunomodulation, TSPO represents a potential target for *Leishmania* chemotherapy. The present study evaluated PK11195, a ligand of this protein, as an anti-leishmanial agent.

**OBJECTIVE:**

To evaluate the leishmanicidal activity of PK11195 against *L. amazonensis* in infected CBA mouse macrophages *in vitro*.

**METHODS:**

The viability of axenic *L. amazonensis*, *Leishmania major*, and *Leishmania braziliensis* promastigotes was assessed after 48 h treatment with PK11195 (0.2-400 µM). Additionally, intracellular parasite viability was evaluated to determine IC_50_ values and the number of viable parasites in infected macrophages treated with PK11195 (50-100 µM). Infected macrophages were then treated with PK11195 (25-100 µM) to determine the percentage of *L. amazonensis*-infected cells and the number of parasites per infected cell. Electron microscopy was used to investigate morphological changes caused by PK11195. The production of free oxygen radicals, nitric oxide, and pro-inflammatory cytokines was also evaluated in infected macrophages treated with PK11195 and primed or not primed with IFN-γ.

**FINDINGS:**

Median IC_50_ values for PK11195 were 14.2 µM for *L. amazonensis*, 8.2 µM for *L. major*, and 3.5 µM for *L. braziliensis*. The selective index value for *L. amazonensis* was 13.7, indicating the safety of PK11195 for future testing in mammals. Time- and dose-dependent reductions in the percentage of infected macrophages, the number of parasites per infected macrophage, and the number of viable intracellular parasites were observed. Electron microscopy revealed some morphological alterations suggestive of autophagy. Interestingly, MCP-1 and superoxide levels were reduced in *L. amazonensis*-infected macrophages treated with PK11195.

**MAIN CONCLUSIONS:**

PK11195 causes the killing of amastigotes *in vitro* by mechanisms independent of inflammatory mediators and causes morphological alterations within *Leishmania* parasites, suggestive of autophagy, at doses that are non-toxic to macrophages. Thus, this molecule has demonstrated potential as an anti-leishmanial agent.

Leishmaniasis, one of the most challenging neglected diseases faced by developing countries, has an estimated incidence of approximately two million new cases per year and afflicts 98 countries and territories, placing ~310 million people at risk of infection (Alvar et al. 2012). Taking this into consideration, together with the increasing number of reported HIV-*Leishmania* co-infections, it is unsurprising that the World Health Organization has labelled this disease a ‘serious public health problem’ (WHO 2010).

Leishmaniasis treatment necessitates the use of highly toxic drugs with extended courses of administration, which sometimes leads patients to abandon treatment, increasing the chance of treatment failure. Moreover, coinfection with HIV has been shown to undermine the effectiveness of available treatments (Croft & Olliaro 2011), making the search for new pharmaceutical compounds an imperative in the ongoing effort to control this disease.

We have previously demonstrated that CBA mouse macrophages can control *Leishmania major* infection, yet are susceptible to *Leishmania amazonensis*, and present different immune-inflammatory profiles in response to *L. amazonensis* and *L. major* infection, indicating the importance of macrophages in controlling infections arising from these parasites (de Souza et al. 2000, Gomes et al. 2003). We have previously used proteomic analysis to identify proteins that are differentially abundant in CBA mouse macrophages infected with either of these *Leishmania* spp., and we hypothesised that some of these proteins could serve as novel targets for chemotherapeutic treatment of leishmaniasis (Menezes et al. 2013).

One of the 162 proteins identified in our proteomic analysis, the translocator protein (TSPO), is associated with a lower relative abundance of peptides in cells infected with *L. amazonensis* compared with those infected with *L. major* (Menezes et al. 2013), possibly indicating that this protein has a role in controlling *Leishmania* infection. TSPO is a component of the peripheral benzodiazepine receptor (PBR), which is composed of three subunits: the 18 kDa isoquinoline-binding protein (IBP), which has been renamed TSPO (Papadopoulos et al. 2006), the 32 kDa voltage-dependent anion channel (VDAC), and the 30 kDa adenine nucleotide transporter (ANT) (McEnery et al. 1992). TSPO is involved in a range of cellular processes, including apoptosis, transport of cholesterol to the mitochondrial matrix, steroidogenesis, cell proliferation, chemotaxis, cellular respiration, and immune response (Veenman et al. 2007). The fact that TSPO is a fundamentally important protein for cell function and maintenance of cellular homeostasis provides compelling evidence for its potential as a chemotherapeutic target. Several specific ligands that modulate TSPO have been described, including benzodiazepines, 1-(2-chlorophenyl)-*N*-methyl-*N*-(1-methylpropyl)-3-isoquinolinecarboxamide (PK11195), and *N,N*-dihexyl-2-(4-fluorophenyl) indole-3-acetamide (FGIN-1-27) (Veenman et al. 2007). Studies have demonstrated that TSPO ligands, including PK11195, may act as agonists or antagonists depending on the ligand concentration and cell type (Totis et al. 1989). Although the underlying mechanisms of action of these TSPO ligands require further clarification, PK11195 and other ligands are currently being used as markers of neuroinflammation in PET imaging (Folkersma et al. 2011), and they also exhibit anticancer (Shoukrun et al. 2008) and immunomodulatory activities (Domingues-Junior et al. 2000). PK11195 has also been shown to increase free radical production in neuronal cells in a TSPO-dependent manner by promoting opening of the mitochondrial permeability transition pore (Jayakumar et al. 2002), and to reduce the proliferation rate of *Plasmodium falciparum* in infected cells (Dzierszinski et al. 2002, Bouyer et al. 2011). However, TSPO ligands have not been tested against trypanosomatids.

The present study aimed to assess the potential antileishmanial effects of a TSPO ligand, PK11195, on CBA mouse macrophages infected with *L. amazonensis* using an *in vitro* model. Considering that infected CBA macrophages are susceptible to *L. amazonensis* and express lower levels of TSPO than those infected with *L. major* (Menezes et al. 2013), we hypothesised that treatment of *L. amazonensis*-infected macrophages with PK11195, in association with host immune response modulation, would induce killing of the intracellular parasites.

## MATERIALS AND METHODS

*Ethics statement* - CBA mice were obtained from the animal care facility at the Gonçalo Moniz Institute (IGM) - FIOCRUZ, housed in pathogen-free conditions, and fed a commercially available diet with water provided ad libitum. All mice were raised under conditions in accordance with the International Guiding Principles for Biomedical Research Involving Animals; all experimental protocols complied with these guidelines, as well as the resolutions established by the Brazilian National Council for the Control of Animal Experimentation (CONCEA). The present study was approved by the Institutional Animal Experimentation Review Board (CEUA) under protocol number 18/2010.

*Anti-leishmanial drug preparation* - The TSPO ligand PK11195 was acquired from Sigma-Aldrich (St Louis, MO, USA), and a 50 mM stock solution was prepared in 100% ethanol (Sigma, St Louis, MO, USA), then aliquoted and stored at -20°C until use. For experimental purposes, this stock solution was diluted into culture medium at varying concentrations. Amphotericin B sodium deoxycholate (Fungizone, Gibco) was purchased from Life Technologies (Carlsbad, CA, USA) as a ready-to-use solution (271 µM).

*Leishmania culture* - Promastigotes of *L. amazonensis* (MHOM/Br88/Ba-125), *L. braziliensis* (MHOM/BR/94/H3456), and *L. major* (MHOM/RI/-/WR-173) were axenically cultured in Schneider's Insect Medium (Sigma, St Louis, MO, USA) supplemented with 50 µg/mL gentamycin (Gibco, Grand Island, NY, USA) and 10% or 20% heat-inactivated foetal bovine serum (Gibco, Grand Island, NY, USA) (Schneider's complete medium). Cultures were maintained in an incubator at 24°C, for no more than six consecutive passages, until parasites reached the stationary growth phase.

*Viability of axenic Leishmania promastigotes* - To determine IC_50_/48 h, axenic promastigotes from stationary cultures of *L. amazonensis*, *L. braziliensis*, and *L. major* were cultivated at a density of 2 × 10^6^ cells/mL in 200 µL Schneider's complete medium in 96-well plates at 24°C. Parasites were treated with 12 two-fold serial dilutions of PK11195 at concentrations of 400, 200, 100, 50, 25, 12.5, 6.25, 3.13, 1.56, 0.78, 0.39, and 0.20 µM, or with the diluent (ethanol), for 48 h. Next, AlamarBlue^®^ (Invitrogen, Carlsbad, CA, USA) cell viability reagent was added to the parasite cultures to a final concentration of 10% v/v, and the cultures were incubated at 24°C for 4 h. Reagent absorbance at wavelengths of 570 and 600 nm was measured using a spectrophotometer (SpectraMax 340 PC, Molecular Devices, Sunnyvale, CA, USA). All experiments were performed in triplicate and individually repeated at least four times.

*Macrophage cultivation* - Peritoneal washing was performed to harvest macrophages from CBA mouse cavities injected with 3% sodium thioglycolate (Sigma, St Louis, MO, USA). All cells were cultured according to the protocol described by Gomes et al. (2003). The peritoneal lavage was first centrifuged at 300 × *g*, then resuspended in complete Dulbecco's modified Eagle's medium (DMEM) (Gibco, Grand Island, NY, USA) supplemented with 20 mM HEPES (Sigma, St Louis, MO, USA), 42 mM sodium bicarbonate (Sigma, St Louis, MO, USA), 10% foetal bovine serum (Gibco, Grand Island, NY, USA), 2 mM glutamine (Gibco, Grand Island, NY, USA), and 10 µg/mL ciprofloxacin (Isofarma, Precabura, CE, BR). Next, cells were plated and incubated at 37°C in a 5% CO_2_, 95% humidity atmosphere for 4 to 6 h, then washed to remove any non-adherent cells. Cells were maintained in 1 mL complete DMEM for further experiments.

*Macrophage viability* - The AlamarBlue^®^ assay was used to determine the cytotoxic concentration 50 (CC_50_) of PK11195 in uninfected macrophages. Uninfected thioglycolate-elicited peritoneal macrophages were cultivated at a density of 2 × 10^5^ cells/mL in 200 µL complete DMEM in 96-well plates at 37°C, and then treated with PK11195 for 48 h at concentrations of 400, 200, 100, 50, 25, 12.5, 6.25, 3.13, 1.56, 0.78, 0.39, and 0.20 µM, in parallel with cultures incubated with the diluent (ethanol) as a negative control. Next, AlamarBlue^®^ was added to the macrophage cultures to a final concentration of 10% v/v, and the plates were then incubated at 37°C for an additional 4 h. Reagent absorbance was measured as described above in *Viability of axenic Leishmania promastigotes*. All experiments were performed in triplicate and individually repeated at least four times.

*Infection and treatment* - To evaluate the anti-leishmanial effect of the TSPO ligand PK11195 on intracellular *L. amazonensis* parasites, CBA mouse macrophages were infected with stationary-phase promastigotes of *L. amazonensis* at a ratio of 10:1 for 6 h. The cells were then washed with saline to remove any non-internalised promastigotes. To assess the effect of PK11195 on intracellular parasites at early stages of infection, macrophages were subsequently treated with PK11195 at concentrations of 25, 50, 75, or 100 µM for 6, 24, or 48 h, whereas control cells were incubated in complete DMEM containing ethanol as a diluent. Additionally, a second control group remained untreated. To evaluate the treatment at later stages of infection, cells that had been washed to remove any non-internalised promastigotes were incubated in fresh DMEM medium for a further 96 h, a period of time sufficient to ensure that all parasites had completed the transformation to the amastigote form inside peritoneal macrophages, as previously shown by Courret et al. (2001). Infected cells were then treated with 50 or 75 µM PK11195, or 2.1 µM amphotericin B sodium deoxycholate, for an additional 24, 48, or 72 h, whereas control cells were incubated with ethanol.

To determine the percentage of infected cells and the number of parasites per infected macrophage at early stages of infection, all cells were fixed and stained with haematoxylin and eosin (H & E). Cell counts were determined by counting no less than 400 cells in random fields under light microscopy at 1000× magnification.

To assess inhibitory concentration 50 (IC_50_) values for intracellular parasites at early stages of infection, CBA mouse macrophages were infected with stationary-phase promastigotes of *L. amazonensis* at a ratio of 10:1 for 6 h. Cells were then washed with saline to remove non-internalised parasites and treated with PK11195 at concentrations of 6.25, 12, 25, 50, 75, 100, 125, 150, and 175 µM for 48 h, whereas a control group remained untreated. All cells were then fixed and stained with 4ʹ,6-diamidino-2-phenylindole (DAPI). The percentage of infected cells was determined by counting cells under fluorescence microscopy as described above. All experiments were performed in quintuplicate and individually repeated at least twice.

Next, the effect of the TSPO ligand on intracellular parasite viability was assessed at early and later stages of infection by measuring the number of viable intracellular parasites in treated and untreated cells as described below in *Intracellular parasite viability*.

*Intracellular parasite viability* - To assess the effect of PK11195 on intracellular parasite viability at early and latest stages of infection, 2 × 10^5^ thioglycolate-elicited peritoneal macrophages from CBA mice were cultivated and infected as described above in *Infection and Treatment*. After treatment, the cells were washed and the medium was replaced with Schneider's complete medium to release amastigotes, which, if viable, later transformed into promastigotes. Finally, the cells were incubated at 24°C for five days, and viable promastigotes were counted in a Neubauer chamber.

*Reversibility of the effect of PK11195 treatment* - To evaluate whether the effect of the TSPO ligand on parasite viability was reversible, thioglycolate-elicited peritoneal macrophages were infected with *L. amazonensis* for 6 h and treated with 75 µM PK11195 for 6, 12, 24, or 48 h. All cells were subsequently washed and incubated with PK11195-free complete DMEM for an additional 48 h, and then the reversibility of the effect of TSPO ligand treatment was assessed by counting the number of viable parasites as described above in *Intracellular parasite viability*.

*Quantification of superoxide production* - The influence of PK11195 on the production of superoxide ions (O_2_^●-^) by NADPH oxidase in the macrophage plasma membrane was assessed using a lucigenin (*N,N*ʹ-dimethyl-9,9ʹ-bisacridinium nitrate)-enhanced chemiluminescence assay to monitor O_2_^●-^ production during phagocytosis. For this assay, O_2_^●-^ production was evaluated in untreated inflammatory peritoneal CBA mouse macrophages and cells pre-treated for 24 h at 37°C with 75 µM PK11195, 500 ng/mL LPS (Sigma, St Louis, MO, USA), or both 75 µM PK11195 and 500 ng/mL LPS.

To quantify O_2_^●-^ production by NADPH oxidase in the plasma membrane, baseline O_2_^●-^ release during phagocytosis was initially measured for 2 min in a luminometer by real-time counting of the number of photons emitted per second due to the reaction of O_2_^●-^ with lucigenin (25 µM) (Sigma, St Louis, MO, USA) prior to the addition of parasites. Next, *L. amazonensis* promastigotes were added, and photon emissions were measured for a further 20 min, after which 2.5 UI/mL of the enzyme superoxide dismutase (SOD, EC 1.15.1.1) was added to convert O_2_^●-^ to H_2_O_2_. The presented results are representative of four independent experiments.

*Quantification of cytokine and NO production* - To assess cytokine production in PK11195-treated cells, 10^6^ thioglycolate-elicited peritoneal macrophages were primed with 50 UI/mL IFN-γ for 24 h and subsequently infected as described above in *Infection and treatment*. Next, infected cells were treated with 50 µM PK11195 for 24 or 48 h in the presence or absence of IFN-γ (R&D Systems, Minneapolis, MN, USA). Culture supernatants were subsequently collected to measure levels of cytokine and nitric oxide (NO) production. Cytokine levels were quantified using a CBA Mouse Inflammation Kit (BD Biosciences) in accordance with the manufacturer's guidelines; this method enables the detection of cytokines in a single tube using antibody-coated beads in a flow cytometric multiplexed bead-based immunoassay. NO production was determined by measuring the accumulation of nitrite in the culture supernatants using the Griess reaction.

*IC_50_, CC_50_, and selectivity index calculations* - After determining parasite (axenic promastigote and intracellular amastigote) and uninfected macrophage viability as described above, IC_50_/48 h and CC_50_/48 h values were calculated using GraphPad Prism software v6.0. Data were normalised and then subjected to nonlinear regression analysis (curve fitting) (de Sá et al. 2009). Selectivity index (SI) values were obtained by calculating the ratio of CC_50_:IC_50_.

*Transmission electron microscopy* - Ultrastructural alterations in infected peritoneal macrophages arising from treatment with the TSPO ligand were assessed by electron microscopy using 75 µM PK11195 for 24 or 48 h. After treatment, all infected macrophages were fixed with Karnovsky fixative (2.5% glutaraldehyde grade II, 2% formaldehyde, and 2.5 mM CaCl_2_ in 0.1 M sodium cacodylate buffer adjusted to pH 7.4) for at least 1 h, and subsequently post-fixed with 1% osmium tetroxide, 0.8% potassium ferrocyanide, and 5 mM calcium chloride in 0.1 M sodium cacodylate buffer, pH 7.4. All cells were subsequently dehydrated using a graded acetone series and embedded in Polybed resin. Ultrathin sections were cut, stained with uranyl acetate and lead citrate, and then examined under a JEM-1230 transmission electron microscope (JEOL USA, Peabody, MA, USA).

*Statistical analysis* - GraphPad v6.0 software was used to perform all statistical analyses. Data were tested for normality using the Shapiro-Wilk normality test to determine whether the obtained results followed a Gaussian distribution. The Student's *t*-test and one-way analysis of variance (ANOVA) were used to evaluate results with normal distributions, whereas the Mann-Whitney and Kruskal-Wallis tests were applied to nonnormally distributed data. Results were considered statistically significant when p < 0.05.

## RESULTS

**Fig. 1:**
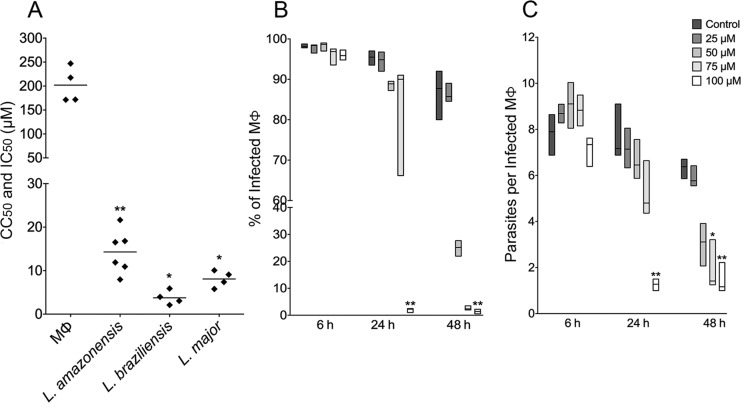
inhibition of the viability of axenic *Leishmania* promastigotes and reduction of parasite load by PK11195. (A) CC50 of macrophages and IC50 of log-phase promastigotes of *L. amazonensis*, *L. braziliensis*, and *L. major* treated with PK11195. Lines represent the mean of four to six independent experiments (symbols) performed in triplicate. Differences between treated and control cells were considered significant when p < 0.05 (Student's t test). (B, C) Drug effects at early stages after infection. Infected macrophages were treated with 400, 200, 100, 50, or 25 μM of PK11195 for different periods of time. The percentage of infected macrophages (B) and the number of parasites per infected macrophage (C) were estimated by cell counting using light microscopy. Lines represent the median and floating bars quartiles (25% and 75%) of four independent experiments performed in quintuplicate (Kruskal-Wallis Test, Dunn's multiple comparison test, *p < 0.05, **p < 0.01).

*PK11195 kills L. promastigotes* - The median IC_50_ value for axenic *L. amazonensis* promastigotes was 14.22 µM [interquartile range (IQR) 10.18-18.02] for parasites treated for 48 h with concentrations of PK11195 ranging from 0.20 to 400 µM (Fig. 1A, Supplementary data, Figure A). PK11195 was observed to have similar effects on the viability of promastigotes of two other *Leishmania* species (*L. braziliensis* and *L. major*) treated for 48 h under identical conditions. The most marked inhibition was observed for *L. braziliensis*, with a median IC_50_/48 h value of 3.51 µM (IQR = 2.34-5.89; Fig. 1A, Supplementary data, Figure B), followed by *L. major*, with a median IC_50_/48 h value of 8.23 µM (IQR = 6.17-9.83; Fig. 1A, Supplementary data, Figure C). Altogether, PK11195 was found to reduce the viability of axenic promastigotes of three *Leishmania* species, which are known to cause cutaneous leishmaniasis, in a dose-dependent manner with low IC_50_/48 h values (Fig. 1A).

The safety of the TSPO ligand was assessed at the same concentrations that were used for treatment of the axenic promastigotes (0.20 to 400 µM), and host cell viability was evaluated using the AlamarBlue^®^ assay. Treatment of macrophages with PK11195 for 48 h led to a decrease in viability, with a corresponding median CC_50_/48 h value of 194.4 µM (IQR = 171.5-239.7; Fig. 1A, Supplementary data, Figure D), giving an SI value of 13.7 for *L. amazonensis*.

*PK11195 reduces parasite infection* - The TSPO ligand also demonstrated an anti-leishmanial effect against intracellular parasites, causing a significant reduction in the percentage of infected macrophages at early stages of infection. A pronounced reduction of 97.75% (IQR = 98.69-97.58) was observed in the percentage of infected macrophages treated with 100 µM PK11195 for 24 h (Fig. 1B). Moreover, a significant reduction in the percentage of infected cells was also observed after 48 h, with a median value of 1.37% (IQR = 0.68-2.05) for cells treated with 100 µM PK11195, compared with 86.88% (IQR = 81.5-90.94) for the control group (Fig. 1B).

**Fig. 2:**
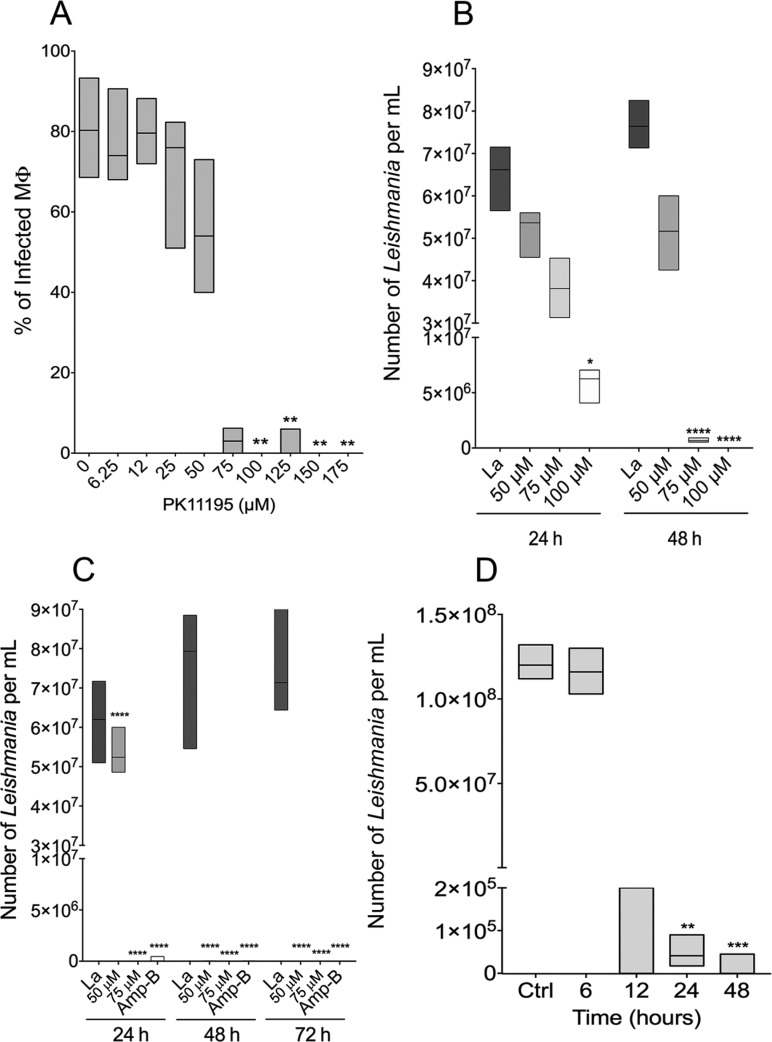
effect of PK11195 on intracellular *Leishmania amazonensis* parasites in infected macrophages. (A) Percentage of infected macrophage. Infected macrophage were treated with varying concentrations of PK11195 for 48 h in order to calculate intracellular parasites IC50/48 h. All experiments were performed in quintuplicate and independently repeated twice. (B) Intracellular parasite viability at the early stages of infection. Macrophages were infected for 6 h and then treated with PK11195 for 24 h or 48 h.(C) Intracellular parasite viability at the later stages of infection. Macrophages were infected for 96 h and then treated with PK11195 for 24 h, 48 h, or 72 h. At each treatment time point, the effect of PK11195 treatment was compared with the effect of amphotericin B sodium deoxycholate treatment. (D) Reversibility of the effect of treatment with PK11195 on the viability of intracellular *Leishmania* parasites. Macrophages were infected for 6 h and treated with 75 μM PK11195. After 6, 12, 24, or 48 h of exposure, macrophages were washed and incubated in PK11195-free complete medium for an additional 48 h, and then the reversibility of the effect of treatment was assessed by counting the number of viable parasites. Lines represent the median and floating bars quartiles (25% and 75%) for independent experiments performed three times in at least in triplicate (Kruskal-Wallis test, Dunn's multiple comparison test, *p < 0.05, **p < 0.01, ***p < 0.001, ****p < 0.0001).

Similar reductions were also seen in the number of parasites per infected macrophage, with median values of 1.41 parasites per macrophage (IQR = 1.28-2.77) and 1.16 parasites per macrophage (IQR = 1.00-2.22) at PK11195 concentrations of 75 and 100 µM, respectively, compared with 6.37 parasites per macrophage (IQR = 5.95-6.65) for the control group (Fig. 1C). The IC_50_/48 h value pertaining to the effect on intracellular amastigotes of treatment with PK11195 at concentrations ranging from 6.25 to 175 µM was 46.55 ± 11.88 µM (Fig. 2A, Supplementary data, Figure E), almost four times lower than the CC_50_/48 h value obtained in macrophages (Fig. 1A).

The effect of PK11195 on parasite load was also assessed at early and later stages of infection by comparing the viability of intracellular parasites in PK11195-treated macrophages and ethanol-treated macrophages. At early stages of infection, cells treated with 100 µM PK11195 showed a significant reduction (91.08%) in the number of live intracellular parasites detected after treatment for 24 h (Fig. 2B). Moreover, treatment with PK11195 for 48 h resulted in more pronounced reductions in the number of viable intracellular parasites: 99.09% and 100% in cells treated with 75 µM and 100 µM, respectively (Fig. 2B).

Macrophage cultures were then treated with PK11195 for 24 h and 48 h after a long-term incubation period of 96 h to ensure that all *L. amazonensis* parasites had completed the intracellular transformation into amastigotes. We observed a 100% reduction in the number of viable intracellular parasites in cells treated with 75 µM PK11195 for 48 h and in cells treated with either 50 or 75 µM of this TSPO ligand for 72 h (Fig. 2C). An identical reduction in the number of viable intracellular parasites was also observed in macrophages treated with 2.1 µM amphotericin B at 48 and 72 h (Fig. 2C).

*Irreversibility of the effect of PK11195 on Leishmania parasites* - Next, we assessed the reversibility of the effect of PK11195 treatment (75 µM) on intracellular parasite survival. A pronounced irreversible reduction in parasite viability of 97.15% (p < 0.001) was achieved as early as 24 h after commencing treatment when infected macrophages were cultured for an additional 48 h following removal of the TSPO ligand. The irreversibility of the effect of PK11195 on parasite viability was found to be 100% (p < 0.0001) after treatment for 48 h (Fig. 2D).

**Fig. 3:**
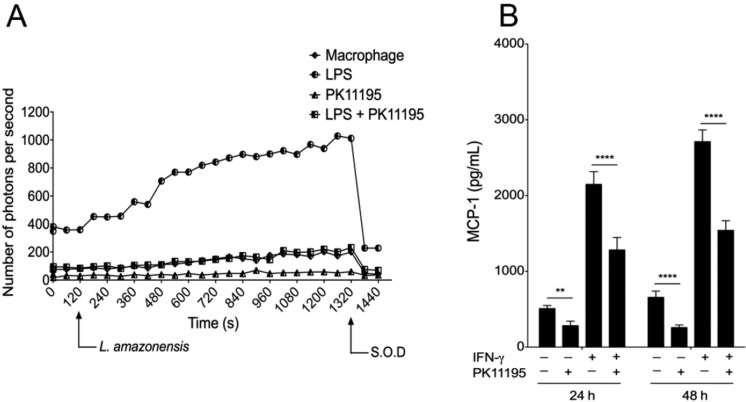
production of inflammatory mediators by macrophages treated with PK11195. (A) NADPH-dependent O2 ^●-^production during phagocytosis. Macrophages were pre-treated for 24 h with PK11195 (75 μM), LPS (500 ng/mL), or both PK11195 (75 μM) and LPS (500 ng/mL). Photon emissions per second were measured prior and after the addition of *Leishmania amazonensis* promastigotes to the culture. Data are derived from one representative experiment out of four independently performed experiments, each with a single replicate (Mann- Whitney test, p = 0.028). (B) MCP-1 production was assessed in cell supernatants of infected macrophages, either primed or not primed with 50 UI/mL IFN-γ for 24 h, and then treated with 50 μM PK11195 for a further 24 or 48 h. Bars represent means ± SD of one representative experiment out of three independently experiments performed in sextuplicate (one-way ANOVA, Sidak's multiple comparisons test **p < 0.01, ****p < 0.0001).

*PK11195 reduces the oxidative response of Leishmania-infected macrophages* - Pre-treatment of macrophages with 75 µM PK11195 prior to *Leishmania* infection resulted in a significant (3.5-4.5-fold) reduction in O_2_^●-^ production by plasma-membrane NADPH-dependent oxidase compared with untreated controls. Similarly, when macrophages were pre-treated with LPS (500 ng/mL), pre-treatment with 75 µM PK11195 resulted in a 5.0-fold reduction in O_2_^●-^ production by plasma-membrane NADPH-dependent oxidase in comparison with positive-control macrophages treated with LPS alone, resulting in O_2_^●-^ levels similar to those produced by untreated control macrophages (Fig. 3A).

*PK11195 reduces MCP-1 production by infected macrophages* - Neither changes in the production of NO nor changes in the levels of the inflammatory cytokines evaluated (IL-6, IL-10, TNF-α, IL-12, IFN-g) were detected in infected macrophages treated with PK11195 for 24 or 48 h. However, PK11195 inhibited production of the chemokine MCP-1 at each time point, with significant reductions in MCP-1 levels observed after 24 h (55.69%, p < 0.0001) and 48 h (39.39%, p < 0.0001) of treatment. This inhibitory effect of PK11195 also occurred in infected macrophages primed with IFN-γ (50 UI/mL), even after treatment for only 24 h (59.69%, p < 0.0001) or 48 h (56.82%, p < 0.0001) (Fig. 3B).

**Fig. 4:**
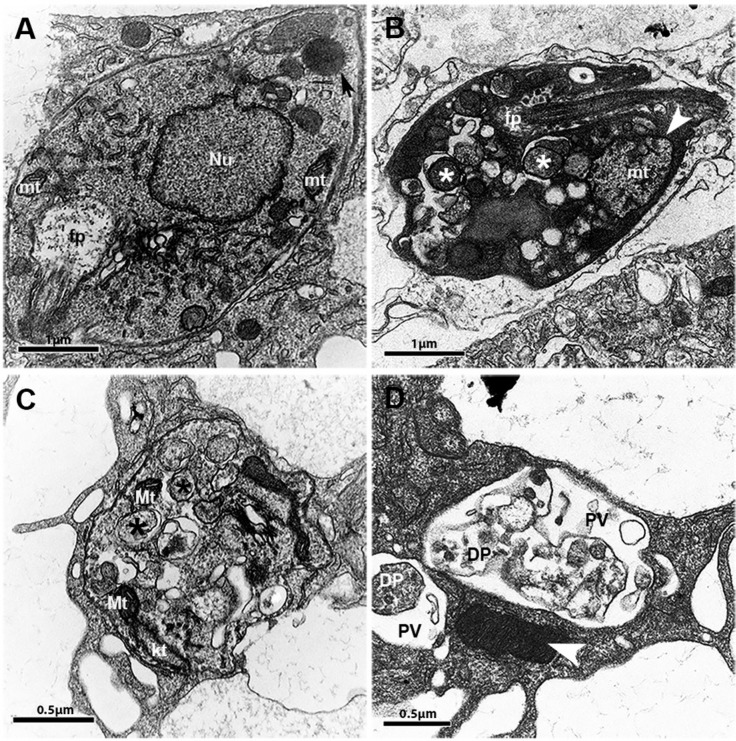
effects of PK11195 on the ultrastructure of *Leishmania amazonensis*. Macrophages were infected and then not treated (A) or treated (B-D) with 75 µM PK11195 for 24 h (B) or 48 h (C and D). Black and white asterisks indicate examples of vacuoles with double membranes, black arrows indicate lipid droplets in normal cells, and white arrowheads indicate mitochondria with increased volume or high electrodensity. Nu: nucleus; kt: kinetoplast; mt: mitochondria; fp: flagellar pocket; PV: parasitophorous vacuoles; DP: dead parasite.

*Ultrastructural alterations in L. amazonensis exposed to PK11195* - Treatment of infected macrophages with PK11195 caused ultrastructural alterations in intracellular parasites suggestive of autophagy induction, including the appearance of double membrane vesicles, compared with parasites within untreated infected control macrophages, Fig. 4A, B, C). In addition, treatment with PK11195 was associated with enhanced mitochondrial size (Fig. 4B), marked cytosolic disorganisation, and the appearance of multivesicular bodies after 24 h (Fig. 4B) and 48 h (Fig. 4C) of treatment; these features were not observed in intracellular parasites within untreated macrophages (Fig. 4A). In addition, debris suggestive of dead parasites was observed inside parasitophorous vacuoles (Fig. 4D), whereas remarkably high electrodensity was observed in the cytosol (Fig. 4B).

## DISCUSSION

The present study, which endeavoured to assess the anti-leishmanial effect of a specific TSPO ligand *in vitro*, found that treatment with PK11195 reduced, in a timeand dose-dependent manner, not only the proliferation of axenic promastigotes and the proportion of infected macrophages in CBA mouse macrophages, but also the number of parasites per infected macrophage and the quantity of viable intracellular parasites. The reduction in axenic promastigote proliferation was observed to be generic, as similar results were seen in species causative of tegumentary leishmaniasis belonging to subgenera *Leishmania* (*L. amazonensis* and *L. major*) and *Viannia* (*L. braziliensis*) (Banuls et al. 2007). With IC_50_/48 h values of 14.22 µM for *L. amazonensis*, 3.51 µM for *L. braziliensis*, and 8.23 µM for *L. major*, which are lower than the CC_50_/48 h value of 194.4 µM observed in treated macrophages, PK11195 was found to be capable of inhibiting the growth of all three *Leishmania* species tested (with SI values of 13.67 for *L. amazonensis*, 55.38 for *L. braziliensis*, and 23.62 for *L. major*), indicating the potential of PK11195 as a chemotherapeutic agent for treatment of cutaneous leishmaniasis.

Although PK11195 exhibits high-affinity binding to TSPO with a reported binding affinity of 9.3 nM (Selleri et al. 2001), its therapeutic anti-parasitic effects (Dzierszinski et al. 2002, Bouyer et al. 2011) and cytotoxic effects on a variety of cancer cell types (Shoukrun et al. 2008) have been shown to occur only at elevated (micromolar) concentrations. The fact that PK11195 exhibited therapeutic effect only at higher concentrations may be explained by the hydrophobicity of PK11195, which is known to bind other proteins, such as alpha-1-acidglycoprotein, which it binds with a high affinity (Lockhart et al. 2003), and albumin, which it binds with low affinity (Dougherty et al. 2002, Lockhart et al. 2003). Our current findings and previous report (Lockhart et al. 2003) support the notion that PK11195 must be employed at relatively high concentrations or be associated with a delivery system (Vaghela et al. 2017) to exert therapeutic effects (Tanimoto et al. 1999).

PK11195 has been used as a marker of cerebral lesions (Folkersma et al. 2011) and as an immunomodulator (Zavala & Lenfant 1987), and has been considered, owing to its pro-apoptotic properties, as a potential chemotherapeutic anticancer agent (Shoukrun et al. 2008). Nonetheless, the literature contains scarce reports on the use of PK11195 as an anti-parasitic molecule. Dzierszinski et al. (2002) demonstrated that PK11195 reduces the proliferation of *P. falciparum* and *Toxoplasma gondii in vitro*. A study by Bouyer et al. (2011) found a reduced proliferation rate of *P. falciparum* parasites in infected erythrocytes treated with PK11195, showing results similar to those described by Dzierszinski et al. (2002). The effects of PK11195 on these parasites from the protozoan phylum Apicomplexa (Dzierszinski et al. 2002, Bouyer et al. 2011) and on trypanosomatids, as demonstrated in the present study, indicate that PK11195 could potentially have an anti-parasitic effect on a wide range of protozoan species.

The direct effect of PK11195 on axenically cultured promastigotes of *Leishmania* cannot be attributed to interaction of PK11195 with TSPO or homologues of TSPO, because there is no evidence that TSPO or homologues of TSPO are present within the *Leishmania* genome. Recently, Hatty et al. (2014) showed that PK11195 interacts with lipids and is incorporated into lipid bilayers, and that incorporation of PK11195 into lipid bilayers alters membrane fluidity. This finding leads us to speculate that the leishmanicidal effect of PK11195 may be in some way associated with alterations in the dynamic properties of the parasite plasma membrane; this possibility deserves further investigation. In addition, it has already been demonstrated that PK11195 has functional effects that are independent of interaction with TSPO (Hatty et al. 2014). However, the exact mechanisms by which this TSPO ligand acts against *Leishmania* infection require further investigation.

In contrast with the observations of Jayakumar et al. (2002) that PK11195 increases free radical productions on neuronal cells, in the present study, PK11195 caused a reduction in the release of O_2_^●-^ by plasma-membrane NADPH oxidase. Our observations are similar to those of Zavala and Lenfant (1987), who demonstrated that production of O_2_^●-^ by P388D_1_ macrophages was attenuated by treatment with arachidonic acid. In addition, we observed that treatment of infected CBA macrophages with PK11195 significantly reduced production of the macrophage attractant chemokine MCP-1; this finding is consistent with a report published by Bribes et al. (2003), which showed that treatment of MRL/1pr mice with PK11195 reduced the amount of inflammatory infiltrate in a mouse model of pulmonary inflammation. In the future, it will be necessary to use an *in vivo* model of *Leishmania* infection to evaluate whether treatment with PK11195 reduces both O_2_^●-^ and MCP-1 levels and, as a result, dampens the inflammatory response via reduction of O_2_^●-^ toxicity and inflammatory cell recruitment, respectively. This effect could be particularly beneficial in treating lesions resulting from *L. braziliensis* infection, in which ulceration and intense inflammation are observed.

The morphological alterations seen in intracellular *L. amazonensis* promastigotes following treatment with PK11195 suggest that cell death may have occurred via multiple mechanisms. The observed swelling of mitochondria and kinetoplasts is suggestive of apoptosis or necrosis, whereas the appearance of double-membrane vacuoles containing degraded material, the presence of multivesicular bodies, and the presence of vesicles in flagellar pockets are alterations suggestive of autophagy. Similar alterations have been described in *L. amazonensis* (Rodrigues et al. 2005), *Trypanosoma cruzi* (Braga et al. 2004), and *Leishmania infantum* (Granthon et al. 2007) treated either with 3-(biphenyl-4-yl)-3-hydroxyquinuclidine, a potent inhibitor of squalene synthase, which is a key enzyme in the metabolism of ergosterol (Granthon et al. 2007), or with ketoconazole, another drug that affects the availability of protozoan ergosterol (Vannier-Santos et al. 1995). In the present study, ultrastructural analysis showed that the intracellular parasites also exhibited double-membrane vesicles, marked cytosolic disorganisation, and the presence of multivesicular bodies, in addition to remarkably high electrodensity in the cytosol and increased amounts of debris suggestive of dead parasites.

Although relatively high doses of PK11195 are required to treat *Leishmania* infection *in vitro*, these levels are much lower than those that cause toxicity to macrophages. In conclusion, the present *in vitro* study has demonstrated the potential of PK11195 as an anti-leishmanial candidate. Further studies are required to evaluate the activity of this TSPO ligand *in vivo*.
