# Supplementary material for: In vitro evaluation of the anti-leishmanial activity and toxicity of PK11195
Source: Mem Inst Oswaldo Cruz. 2018 Feb 5;113(4):e170345. doi: 10.1590/0074-02760170345 (PMC5851033; doi:10.1590/0074-02760170345)
Supplement: Supplementary file 1 [file 0074-0276-mioc-113-4-e170345-Suppl01.pdf]

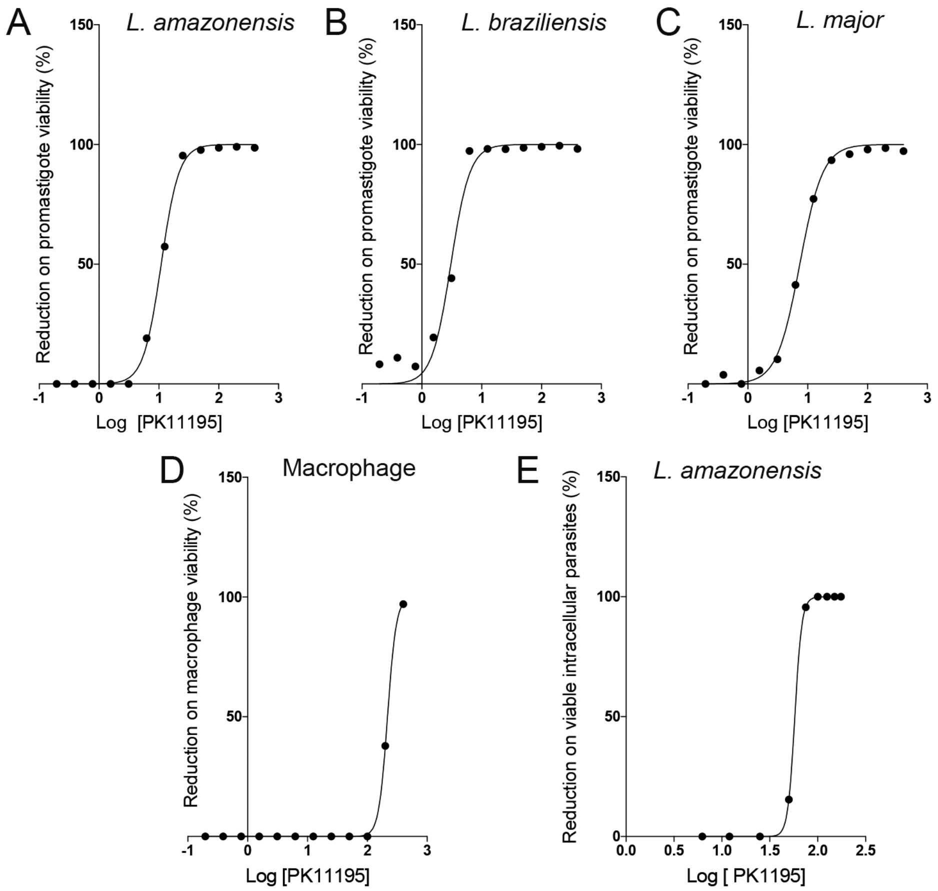

Effect of PK11195 on macrophage and *Leishmania* viability. Log-phase promastigotes of (A) *L. amazonensis*, (B) *L. braziliensis*, and (C) *L. major*, as well as (D) macrophages, were treated for 48 h with 12 two-fold serial dilutions of PK11195 at the following concentrations: 400, 200, 100, 50, 25, 12.5, 6.25, 3.13, 1.56, 0.78, 0.39, and 0.20 µM. Macrophage and parasite viabilities were measured using the AlamarBlue® assay. (E) To determine intracellular parasite viability, macrophages infected with parasites at a 10:1 ratio were treated with 25, 50, 75, or 100 µM PK11195 for 48 h. At the end of the incubation time, all groups of cells were fixed and stained with DAPI, and at least 400 cells were counted in random fields under fluorescence microscopy.
